# Successful Radiofrequency Lesioning of the Pallidothalamic Tract in Paretic Form Focal Hand Dystonia With Paradoxical Unexpected Response to Intraoperative Test Electrical Stimulation: A Case Report

**DOI:** 10.7759/cureus.68088

**Published:** 2024-08-29

**Authors:** Yoji Kuramoto, Takaomi Taira, Saujanya Rajbhandari, Shinichi Yoshimura

**Affiliations:** 1 Neurosurgery, Hyogo Medical University, Nishinomiya, JPN; 2 Functional Neurosurgery, Kumagaya General Hospital, Kumagaya, JPN; 3 Diagnostic and Neuroradiology, Bern University Hospital, Bern, CHE

**Keywords:** paradoxical unexpected response, intraoperative test electrical stimulation, pallidothalamic tract, focal dystonia, lesioning in dystonia, stereotactic surgery, paretic form dystonia

## Abstract

We report a case with paretic focal hand dystonia, which at first glance was diagnosed as writer's cramp, with poor performance only when playing the guitar and writing but with increased muscle tension around the elbow rather than in the fingers and hands. The muscle tension was around the elbow and the pallidothalamic tract (PTT) was selected as the proximal muscle target with less permanent complications. During the operation, the PTT test electrical stimulation was impaired only for guitar playing, but not for other hand movements. Therefore, test lesioning at a lower temperature and for a shorter time improved the symptoms, so we were convinced that this was the target site and coagulated this site, i.e., the PPT, at the usual temperature and time. With only one target lesioning, the patient's symptoms disappeared for six months. Careful history taking and physical examination to identify the site of muscle tension is important in determining the target of paretic form dystonia. In addition, test lesioning at a lower temperature and for a shorter time is useful if the test electrical stimulation produces a paradoxically unexpected response.

## Introduction

Paretic form dystonia is a disorder in which the patient feels tired and weak in holding a pen, although there is no obvious abnormal posture or involuntary movement [1]. In this report, the diagnosis was of a patient with paretic form focal hand dystonia who complained of poor performance only when playing the guitar and writing. However, when the patient had difficulty using his hands, there was no muscle tension in the hands, but in the muscles around the elbow, which are proximal to the hands, and this muscle tension was considered to be causing the symptoms. Intraoperative test electrical stimulation of the pallidothalamic tract (PTT) only worsened task-specific symptoms, contrary to our expectations. We considered that the movement-specific effects of the test stimulation were sites where the PTT was associated with paretic form dystonia. To prove this, test lesioning of the PTT was performed at low temperatures and for a short time. This test lesioning released his symptoms. The methods for identifying target sites in such paretic form dystonia and how to respond to paradoxically unexpected findings from intraoperative test electrical stimulation will also be shared.

## Case presentation

A male in his 20s had difficulty playing the guitar six years ago. Two years ago, he visited our hospital due to elevation and abduction of his right shoulder. Trihexyphenidyl was not effective; therefore, he received a one-time Botox injection to his trapezius and supraspinatus muscles. After the Botox injection, his shoulder symptoms disappeared, but the following two symptoms appeared: first, when he played the guitar quickly, the right-hand pick was not stable. Second, he could not write neatly in small letters or long sentences. At a glance, symptoms were motion-specific and were found only in the hands. Initially, we hypothesized that Botox had resolved the periarticular shoulder dystonia, with the delayed manifestation of writer's cramp (focal hand dystonia). Further history taking and physical examination revealed muscle tension observed in the triceps brachii, biceps brachii, and pronator teres muscles around his right elbow during guitar playing, but no muscle tension in the hand. When writing small letters, his fingers had difficulty moving smoothly without hyperactivity of the wrist and hand (Video 1).

**Video 1:** Preoperative video The hand was shaking slightly at the start of writing. Muscle hyperactivity was noted in the proximal forearm (author's finger-pointing). When playing guitar, the patient had difficulty holding the pick, and muscle hyperactivity was noted in the biceps and triceps muscles.

The symptoms were mainly difficulty in use without muscle tension and were considered paretic form focal hand dystonia. The movement and disability scales of the Burke-Fahn-Marsden Dystonia Rating Scale (BFMDRS) were 2 points each, the Arm Dystonia Disability Scale (ADDS) was 60 points, and Tubiana and Chamagne dystonia scale for musician's dystonia (TCS) was stage 2. Muscle tension around the elbow was considered to inhibit the drive of the fine muscle movement of the hand. Therefore, radiofrequency (RF) lesioning of the PTT was performed to control the proximal muscle tension.

Stereotactic surgery was performed using the Leksell Stereotactic System (Elekta, Stockholm, Sweden). Stereotactic planning was done using the StealthStation S8 system (Medtronic, Dublin, Ireland). The tentative target was set at left: 9 mm, and inferior: 1 mm from the mid-commissural point. After insertion of a lesioning needle (No. 1017044, Elekta, Stockholm, Sweden) with a width of 1 mm and a length of 4 mm at the coagulation site at the needle tip, test electrical stimulation was performed at a current of 3 mA, frequency of 130 Hz, and pulse width of 100 μs. During test electrical stimulation, he only had difficulty playing the guitar. However, other movements were fine, and no numbness or dysarthria appeared. Based on this finding, the PTT was determined to be the relevant region. To prove that this site is related to the symptoms, RF lesioning at a lower temperature than usual, i.e., 65°C, and for a shorter period of 30 seconds as a test coagulation, with the guitar playing, immediately improved the guitar performance. Therefore, we added lesioning at 70°C for 30 seconds. Symptoms during writing also improved (Video 2).

**Video 2:** Intraoperative video. The patient was unable to play the guitar when the test electrical stimulation was done with a lesioning needle placed in the pallidothalamic tract (PTT). No problems were found in other movements. After a test lesioning at 65°C for 30 seconds, his guitar playing became good. Lesioning at 70°C for 30 seconds was added. Symptoms during writing also disappeared.

**Figure 1:**
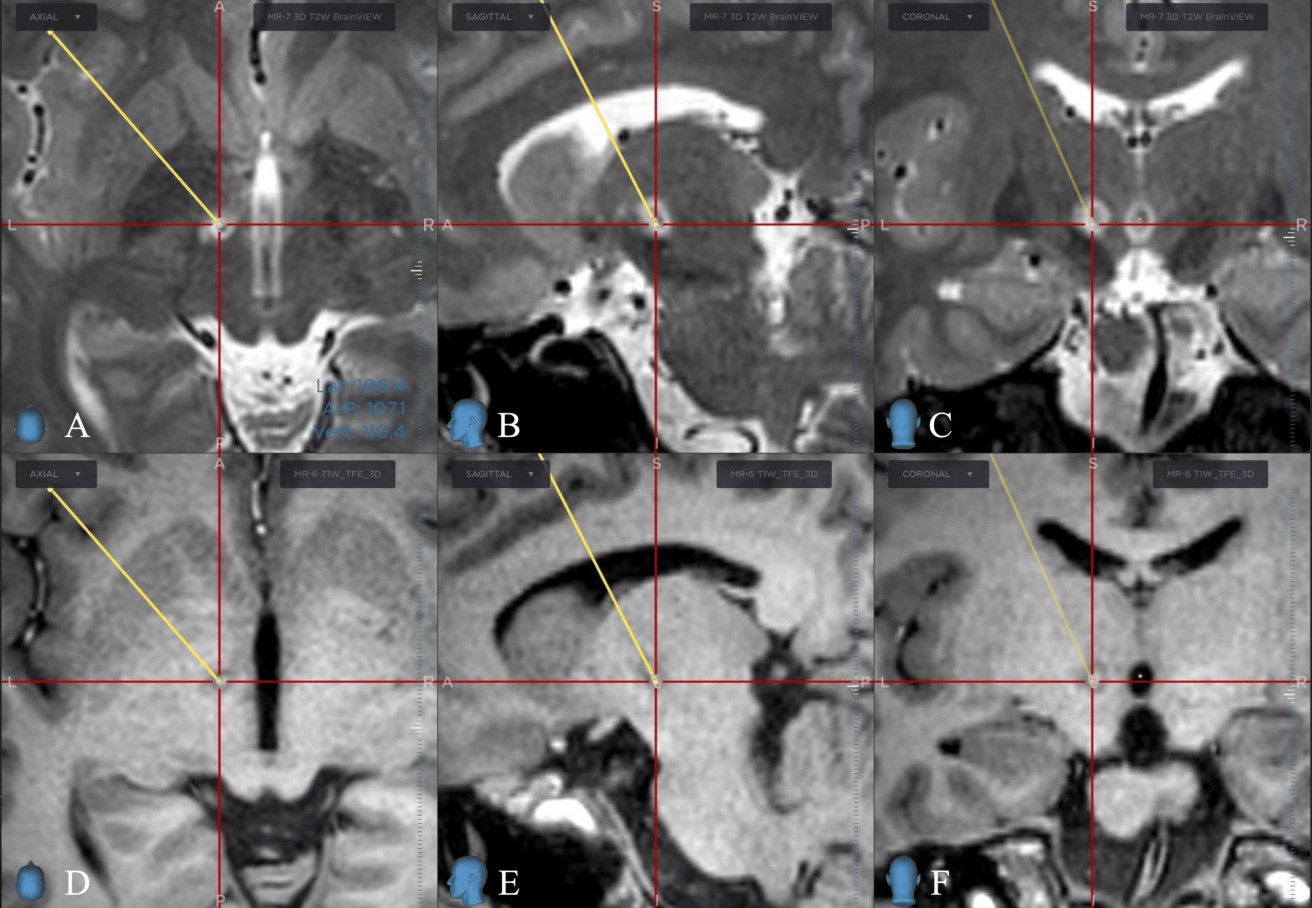
Postoperative MRI. The planned target and the coagulation point are almost in the same location. (A) Axial, (B) sagittal, and (C) coronal sections on T2-weighted imaging (T2WI). (D) Axial, (E) sagittal, and (F) coronal sections on T1-weighted imaging (T1WI). The yellow line shows the planned trajectory, and the intersection of the red lines, i.e., the center of each panel, is the target set preoperatively.

Postoperative MRI confirmed the accurate site of lesioning (Figure 1).

He was discharged from our hospital two days after this surgery without complications. Improvement was maintained at three and six months, with a BFMDRS of 0, ADDS of 100, and TCS of stage 4.

## Discussion

We presented a case of paretic form focal hand dystonia, apparently diagnosed as writer's cramp, which was treated with RF lesioning of the PTT and showed improvement. In paretic form dystonia, the patient feels fatigued and weak with certain movements, despite the absence of obvious abnormal posture or involuntary movements [1]. This type of dystonia is sometimes referred to as negative dystonia [2], but this is debatable. If we were to consider this patient as simply a writer's cramp patient, we would have chosen stereotactic RF lesioning or deep brain stimulation (DBS) to ventro-oral nucleus (Vo), which is used for medically refractory focal hand dystonia [3]. However, we considered the target to Vo unsuitable in this case because muscle tension was observed in the muscles around the elbow, not in the finger and hand based on a detailed medical history and physical examination. Therefore, we hypothesized that hand fine motor control was impaired by muscle tension in the proximal muscles around his right elbow. The target in upper arm and peri-shoulder dystonia is the same as in neck and trunk dystonia, with the most common target being the globus pallidus internus (GPi). GPi has long been used as a target for proximal muscle dystonia [4,5]; however, about 2% after pallidotomy developed lacunar infarction [6,7]. Recently, we chose PTT as an alternative target to GPi [8].

The PTT, which connects the GPi to the thalamus, consists of ansa lenticularis and lenticular fasciculus, starts from the GPi, divides into two parts, and runs medially. They join in the space between the subthalamic nucleus and the mammillothalamic tract. This confluence is called the Forel H field. After that, these fibers head toward the thalamus. The area from this confluence to the thalamus is called the Forel H1 field and this is exactly where we coagulated [9]. In the past, the PTT was a target for intractable epilepsy [10]. Recently, the PTT was re-evaluated as a target for dystonia, epilepsy, and obsessive-compulsive disorder [8,11,12].

Intraoperative findings were unexpected: only guitar playing was difficult when the PTT was stimulated by electrical testing; other simple movements were possible without problems. Intraoperative test electrical stimulation often improves symptoms if the site is appropriate. In DBS, intraoperative test electrical stimulation has also been reported to contribute to improved target accuracy [13]. We had experienced this paradoxical response with test stimulation even when the needle tip is appropriately positioned, although this is rare. If other movements than playing the guitar were also poor during the test electrical stimulation, the stimulation could have affected the internal capsule, and we hesitated to coagulate this point. But other movements were not affected at all, the stimulation led us to believe that this movement specificity was related to this disease. We empirically knew that even if the needle tip was appropriate, such paradoxical reactions could sometimes occur. To solve this problem, we performed test coagulation at a lower temperature, and for a shorter time, which is usually performed at 70°C for 40 seconds. The improvement in symptoms after the test electrical stimulation confirmed that our hypothesis about the disease is correct. This case illustrates the importance of considering the mechanism, whether it is the patient's symptoms or the response to the test electrical stimulation, by careful observation and checking for other symptoms, rather than making judgments based solely on the apparent symptoms.

## Conclusions

We experienced a case of paretic form focal hand dystonia caused by proximal muscle tension that was improved by lesioning of the PTT. It was important to identify areas of muscle tension with careful history-taking and physical examination. In addition, careful observation of symptoms elicited during the test of electrical stimulation could be helpful in treatment. If a target lesion is suspected, especially if the response to test electrical stimulation is contrary to expectations, a lower temperature and a shorter test lesioning could prove useful, especially if it is difficult to determine whether the site is a coagulation site or not.
